# Detection of three closely located single nucleotide polymorphisms in the EAAT2 promoter: comparison of single-strand conformational polymorphism (SSCP), pyrosequencing and Sanger sequencing

**DOI:** 10.1186/1471-2156-15-80

**Published:** 2014-07-05

**Authors:** Shavanthi Rajatileka, Karen Luyt, Maggie Williams, David Harding, David Odd, Elek Molnár, Anikó Váradi

**Affiliations:** 1 Centre for Research in Biosciences, Department of Biological, Biomedical and Analytical Sciences, Faculty of Health and Applied Sciences, University of the West of England, Bristol BS16 1QY, UK; 2 Neonatal Neuroscience, School of Clinical Sciences, University of Bristol, St Michael’s Hospital, Southwell Street, Bristol BS2 8EG, UK; 3 Bristol Genetics Laboratory, Pathology Sciences, Blood Sciences and Bristol Genetics, Southmead Hospital, Bristol BS10 5NB, UK; 4 Regional Neonatal Intensive Care Unit, St Michael’s Hospital, University Hospitals NHS Trust, Bristol BS2 8EG, UK; 5 Neonatal Intensive Care Unit, Southmead Hospital, North Bristol NHS Trust, Bristol BS10 5NB, UK; 6 School of Physiology and Pharmacology University of Bristol, Medical Sciences Building, University Walk, Bristol BS8 1TD, UK

**Keywords:** *EAAT2* promoter, Single nucleotide polymorphism, Genotyping, Pyrosequencing, SSCP, Premature newborns, Dried blood spots, Glutamate regulation

## Abstract

**Background:**

Single-strand conformational polymorphism (SSCP) is still a frequently used genotyping method across different fields for the detection of single nucleotide polymorphisms (SNPs) due to its simplicity, requirement for basic equipment accessible in most laboratories and low cost. This technique was previously used to detect rs4354668:A > C (g.-181A > C) SNP in the promoter of astroglial glutamate transporter (*EAAT2*) and the same approach was initially used here to investigate this promoter region in a cohort of newborns.

**Results:**

Unexpectedly, four distinct DNA migration patterns were identified by SSCP. Sanger sequencing revealed two additional SNPs: g.-200C > A and g.-168C > T giving a rise to a total of ten *EAAT2* promoter variants. SSCP failed to distinguish these variants reliably and thus pyrosequencing assays were developed. g.-168C > T was found in heterozygous form in one infant only with minor allele frequency (MAF) of 0.0023. In contrast, g.-200C > A and -181A > C were more common (with MAF of 0.46 and 0.49, respectively) and showed string evidence of linkage disequilibrium (LD). In a systematic comparison, 16% of samples were miss-classified by SSCP with 25-31% errors in the identification of the wild-type and homozygote mutant genotypes compared to pyrosequencing or Sanger sequencing. In contrast, SSCP and pyrosequencing of an unrelated single SNP (rs1835740:C > T), showed 94% concordance.

**Conclusion:**

Our data suggest that SSCP cannot always detect reliably several closely located SNPs. Furthermore, caution is needed in the interpretation of the association studies linking only one of the co-inherited SNPs in the *EAAT2* promoter to human diseases.

## Background

Genetic analysis is one of the fastest-growing areas of clinical diagnostics. Changes to a single nucleotide, known as single nucleotide polymorphism (SNP) is one of the major types of variants identified in the human genome. On average, in the human genome SNPs are distributed at 1 SNP per 1000 base pairs [1,2]. Some of these inherited SNPs play an important role in human diseases, while others are less relevant clinically and are phenotypically silent.

PCR amplification followed by Sanger DNA sequencing is one of the most commonly used methods of identifying SNPs in a sample cohort [3,4]. However, the cost per sample is still relatively high [5] and typically the sequencing run length is ~3 hours (based on genotyping ~700 bp amplicon using capillary array electrophoresis technology) [6]. Due to these drawbacks single-strand conformation polymorphism (SSCP) is still very frequently used across many different fields for SNP detection [7-15]. SSCP is a rapid, reproducible and quite simple method that does not require specialised expensive equipment or reagents. The SSCP process involves PCR amplification of the target fragment, denaturation of the double-stranded PCR product with heat and formamide and electrophoresis on a non-denaturing polyacrylamide gel. During electrophoresis the single-stranded DNA (ssDNA) fragments fold into three-dimensional shape depending on their primary sequence [7]. DNA fragments can then be genotyped as a result of their different migration patterns and then confirmed by Sanger sequencing. SSCP sensitivity varies considerably from 70% to 95% [16-19]. The disadvantages of this technique are that it is relatively labour intensive, low throughput and requires Sanger sequencing of a representative sample cohort to confirm the nucleotide sequence.

Pyrosequencing [20], a non-gel based, real-time, DNA sequencing-by-synthesis technique that is based on the luminometric detection of released pyrophosphate (PPi) during nucleotide incorporation, has also been used extensively for sample genotyping [21-26]. Pyrosequencing relies on a cascade of enzymatic reactions that yields detectable light, which is proportional to the incorporated nucleotides. The resulting pyrograms produce peak patterns in short stretches of the DNA sequence analysed, which vary between genotypes, and can distinguish between the different alleles at a named position. A large number of samples can therefore be analysed in a cost and time effective manner.

In this study, we investigated a previously identified SNP (rs4354668:A > C; [11]) in the promoter of the astroglial glutamate transporter *EAAT2* (SLC1A2) at position -181bp (g.-181A > C) in genomic DNA of newborn infants. The rational for looking at this particular SNP was that previous studies using SSCP found association of this SNP with increased extracellular glutamate levels and neurodegeneration in adult stroke patients [11]; with a higher risk of relapsing multiple sclerosis [27] and the progression of migraines into chronic daily headaches [28]. Unexpectedly, we identified two additional SNPs in the *EAAT2* promoter; g.-200C > A and g.-168C > T. The g.-168C > T SNP was only found in one individual in a heterozygous form in the entire cohort. In contrast, g.-200C > A and g.-181A > C sequence variants were much more common and they were in Linkage Disequilibrium (LD). SSCP was not discriminatory enough to clearly show differences between the various genotypes and 31% of homozygote mutants (mutant/mutant; MT/MT) and 25% wild-type (WT/WT) genotypes were identified incorrectly using this technique when compared to sequencing data. In contrast, pyrosequencing detected all naturally occurring variants in the highly GC-rich region and showed 100% concordance with Sanger sequencing suggesting that it can be used successfully to detect closely positioned and linked SNPs. Our data also indicate that the interpretation of the studies [11] attributing a causal link between g.-181A > C and adult neurological diseases is incomplete as the SNP was potentially misclassified and the LD with another SNPs not considered.

## Methods

### Sample collection and processing

Newborn dried blood spots (DBS) were collected from predominantly Caucasian infants (91.6% white, 8.4% non-white) born in the greater Bristol area (UK) participating in an association study to investigate the genetic background of newborn infants to white matter brain injury. The study received ethical approval in April 2008 from the National Research Ethics Service, UK (REC reference number 10/H0106/10 [29]). Samples, collected from 239 infants within the past 3-22 years, were used in the study. All blood spot screening cards were stored in the biobank in boxes at room temperature. Whole blood samples were collected from nine healthy adult volunteers to optimise protocols used in the study. Genomic DNA was isolated and quantified as we described previously [29].

### PCR amplification of EAAT2 promoter for SSCP analysis

**Figure 1:**
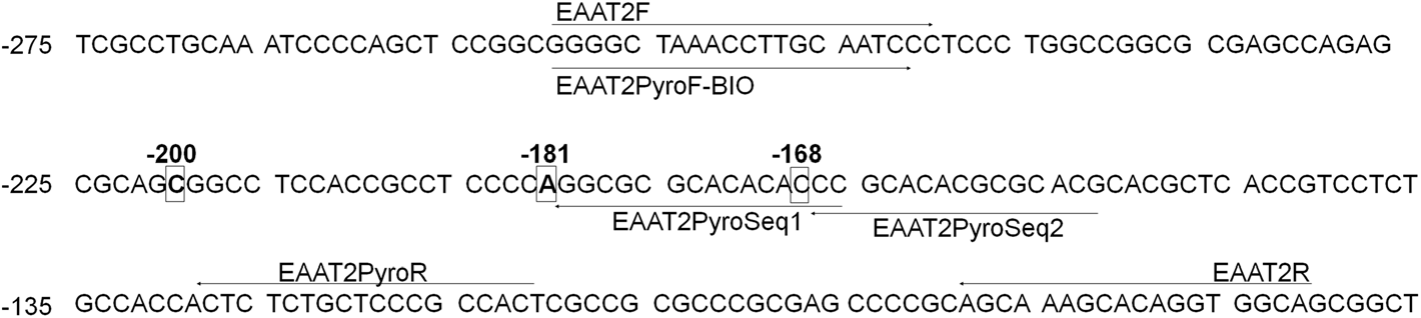
**Promoter sequence of the human *EAAT2 *(Accession AF510107.1).** The primers and the positions of the three SNPs at -200bp (g.-200C > A), -181bp (g.-181A > C) and -168bp (g.-168C > T) are indicated. Numbering is relative to the transcription start site. Primers EAAT2F and EAAT2R were used for standard PCR and Sanger sequencing while EAAT2PyroF-BIO and EAAT2Pyro-R were used to generate biotinylated PCR products and EAAT2PyroSeq1 and EAAT2PyroSeq2 for pyrosequencing (see also Table 1).

Previously described primers EAAT2F and EAAT2R were used to amplify the EAAT2 promoter fragment (GeneBank accession AF510107.1; Figure 1 and Table 1[11]). All PCR reactions were carried out for 35 cycles in a total volume of 25 μl, containing 1× high fidelity reaction buffer - (500 mM KCl, 100 mM Tris-HCl, pH 8.3), 1 mM of MgCl_2_, 200 μM of each dNTP, 100 pmol of each oligonucleotide primer, 1 unit of high fidelity Taq Polymerase (FastStart High Fidelity Taq Polymerase, Roche Diagnostics Limited, West Sussex, UK) and 2 μl (~1-30 ng) of gDNA. Additionally, a final concentration of 1× GC-rich solution (Roche Diagnostics Limited, West Sussex, UK) was added to each reaction. Reaction parameters were 95°C for 5 min followed by 35 cycles of 95°C for 30 s, 60°C for 45 s and 72°C for 1 min. A final extension at 72°C was carried out for 10 min.

**Table 1:** Pyrosequencing primers and conditions used in the study

**Oligonucleotide**	**Sequence 5′-3′**	**Product (bp)**	**Annealing T (°C)**	**Annealing T (°C)**
**EAAT2F**	GGGGCTAAACCTTGCAATCC	180	65	None
**EAAT2R**	CTGCCACCTGTGCTTTGC			
**EAAT2PyroF-BIO**	GGGGCTAAACCTTGCAATC	166	60	5′ Biotin
**EAAT2PyroR**	GAGTGGCGGGAGCAGAGA			None
**EAAT2PyroSeq1**	GGGTGTGTGCGCGCC	N/A		None
**EAAT2PyroSeq2**	CCGCACACGCGCACG	N/A		None
**Target sequence for pyrosequencing (1)**		**T**/**G**GGGGAGGCGGTGGAGGCC**G**/**T**CTG		
**Nucleotide dispensation order (1)**		CGTGCAGCGTGAGCGTGC		
**Target sequence for pyrosequencing (2)**		**G**/**A**TGTGTGCGCGCC		
**Nucleotide dispensation order (2)**		CAGTGTGT		

Primer pair EAAT2PyroF-BIO/EAAT2PyroR were used to generate biotinylated PCR products flanking SNPs g.-200C > A; g.-181A > C and g.-168C > T. Primers EAAT2PyroSeq1 (to detect g.-200C > A;-181A > C) and EAAT2PyroSeq2 (to detect g.-168C > T) were used for pyrosequencing. In the dispensation order the nucleotides used as negative controls are underlined. The nucleotide change in the Target sequence for pyrosequencing is indicated in bold.

### SSCP analysis

SSCP was performed as previously described [8]. PCR samples were resolved on 0.5× acrylamide gels containing 12.5 ml MDE® (Mutation Detection Enhancement) gel solution (Lonza Group Ltd., Basel, Switzerland), 3 ml of 10× TBE (Tris/Borate/EDTA, pH 8.3) buffer, 34.28 ml deionised water, 20 μl tetramethylethylenediamine (TEMED; Sigma-Aldrich, St Louis, Missouri, UK) and 200 μl of freshly prepared 10% ammonium persulfate (APS; Sigma-Aldrich, St Louis, Missouri, UK). PCR samples were prepared for electrophoresis as follows; 3 μl of PCR product was mixed with 7 μl of denaturing loading buffer (95% formamide, 0.025% bromophenol blue, 0.025% xylene cyanol and 20 mM EDTA) (all reagents from Sigma-Aldrich, St Louis, Missouri, UK). The mixture was heated to 95°C for 5 min, rapidly cooled on ice and then 10 μl was loaded and run for 30 min at 300 V. The voltage was then reduced to 150 V and the DNA strands separated for 14 h at room temperature (~20°C). The gel was washed twice in distilled water for 10 s and then incubated in 0.5% glacial acetic acid (Fisher Scientific, Loughborough, UK) and 10% molecular grade ethanol (Sigma-Aldrich, St Louis, Missouri, UK). The gel was then incubated in 0.1% silver nitrate (Sigma-Aldrich, St Louis, Missouri, UK) solution for 20 min and rinsed with distilled water twice. The gel was then washed with developing solution, 1.5% NaOH (Fisher Scientific, Loughborough, UK) and 0.15% molecular grade formaldehyde (Sigma-Aldrich, St Louis, Missouri, UK) for 20 min. The gel was fixed in 0.75% sodium carbonate (Fisher Scientific, Loughborough, UK) solution for 10 min. The DNA bands were visualized on a light box and the samples were scored.

### Generation of biotinylated PCR products for pyrosequencing

Two sequence-specific primers (EAAT2PyroF-BIO and EAAT2PyroR; Figure 1, Table 1) were designed to flank all SNPs in the *EAAT2* promoter using the software provided by Qiagen Pyrosequencing, with the forward primer biotinylated. PCR reactions contained 1× PCR buffer (500 mM KCl, 100 mM Tris-HCl, pH 8.3), 1.5 mM MgCl_2_, 200 μM of each dNTP, 100 pmol of each oligonucleotide and 1 unit of high fidelity Taq polymerase (FastStart High Fidelity Taq Polymerase, Roche Diagnostics Limited, West Sussex, UK) per reaction. Two microlitres of genomic DNA (containing 4-6 ng DNA) was used per reaction. Amplification was performed with the following conditions: 95°C for 5 min; 50 cycles of 94°C for 30 s, 60°C for 30 s and elongation at 72°C for 30 s; followed by the final extension for 10 min at 72°C. Pyrosequencing and Sanger sequencing were carried out as we described previously [29]. The target sequence for analysis and the nucleotide dispensation order for the pyrosequencing assay are shown in Table 1. Purified PCR products were Sanger sequenced using primer EAAT2R (Table 1).

## Results

### Analysis of the EAAT2 promoter using SSCP

**Figure 2:**
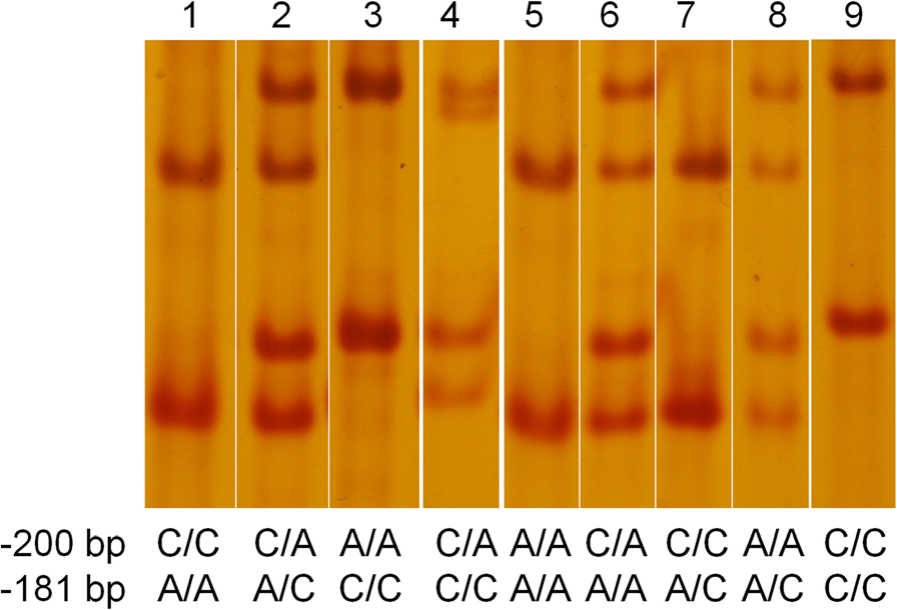
**SSCP patterns of the *EAAT2 *promoter genotypes.** Following PCR amplification, all samples were run on the same SSCP gel and then visualised. The genotype of each sample determined by Sanger sequencing is shown at the bottom of each lane. Note that all these samples were wild type for g.-168C > T.

A SNP was detected in the *EAAT2* promoter at -181bp by SSCP [11]. Since we were interested in this promoter region and already had considerable expertise in this method [8], we used SSCP for our initial experiments. Although it is not possible to predict the three-dimensional structure from the primary sequence of the ssDNA [19], it is expected that the wild-type (WT/WT), mutant (MT/MT) and heterozygote (WT/MT) would have a unique electrophoretic mobility. Indeed, our SSCP result showed the expected three distinct patterns (Figure 2, Lanes 1-3). However, one sample (Figure 2, Lane 4) showed some unexpected extra bands. Sanger sequencing of samples scored based on their migration pattern as wild type (n = 4, Lane 1), heterozygotes (n = 2, Lane 2), homozygote mutants (n = 2, Lane 3) and the sample with an unusual DNA migration (n = 1, Lane 4) revealed a previously unpublished polymorphism C to A transition at -200bp (g.-200C > A), 19 bp upstream from the A to C transition observed at -181 bp (g.-181A > C; [11]).

Sanger sequencing also revealed that the following additional genotypes exist (sequence is given in -200 bp and -181bp order): A/A and A/A (Figure 2, Line 5); C/A and A/A (Figure 2, Line 6); C/C and A/C (Figure 2, Lane 7); A/A and A/C (Figure 2; Line 8); C/C and C/C (Figure 2, Line 9). These variants did not migrate differently compared to the three main types (Figure 2, Lines 1-3), even when the SSCP running conditions were further optimised suggesting that this technique is unsuitable for the detection of all nine possible *EAAT2* variants (Figure 2).

### Optimization of pyrosequencing to detect all EAAT2 variants

**Figure 3:**
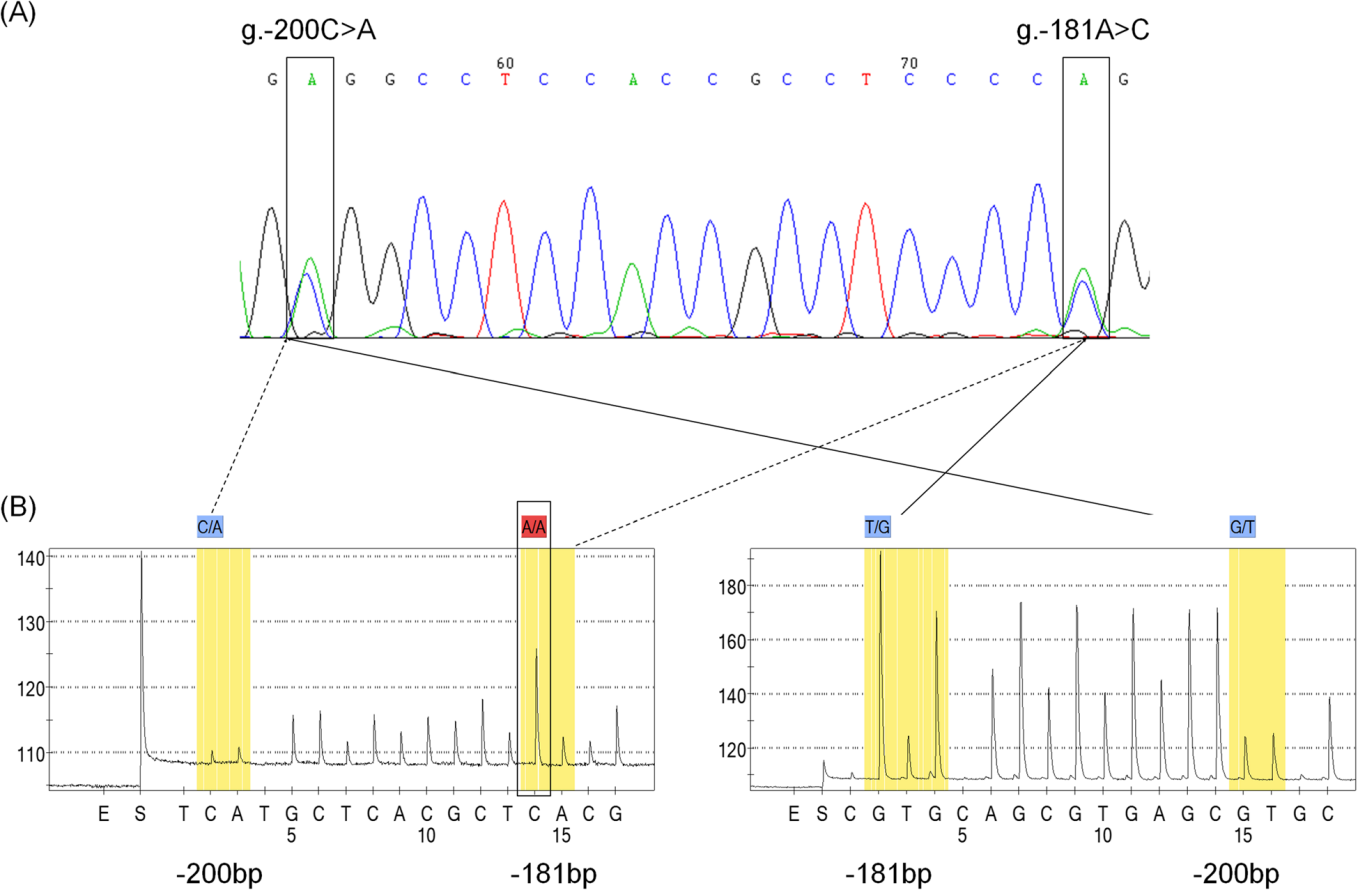
**Pyrograms using forward and reverse strands for sequencing. (A)**. SNPs g.-200C > A;-181A > C are indicated in rectangles on the Sanger sequence traces. **(B)** Pyrograms of the same sample using forward (left panel) and reverse (right panel) primers. Arrows indicate the region sequenced by both methods.

**Figure 4:**
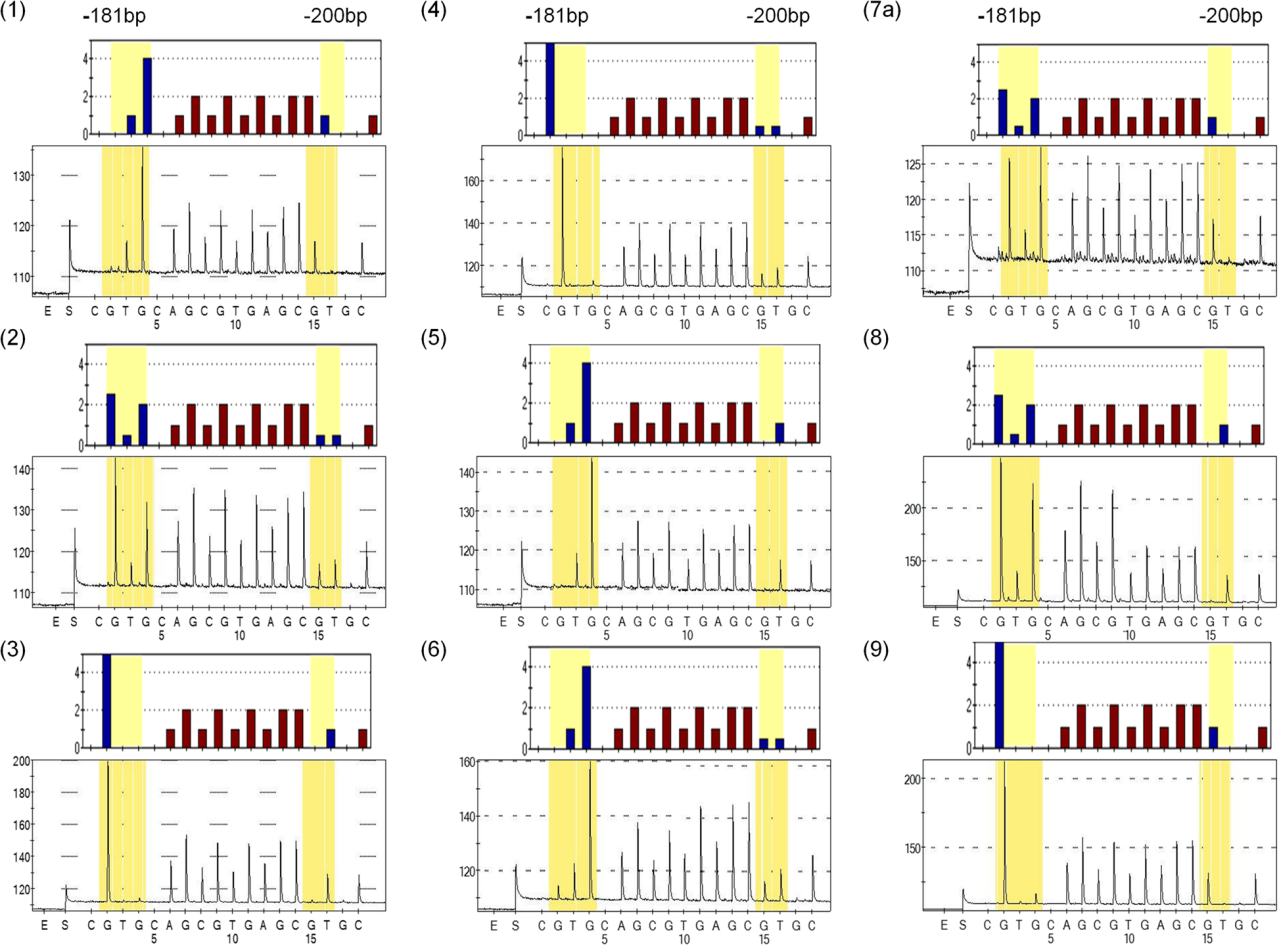
**Predicted (top panels) and observed (bottom panels) pyrograms for *EAAT2 *promoter SNPs.** The position of the SNPs is highlighted in yellow boxes, the x-axis of each pyrogram indicates the order of reagent addition (E - enzyme, S -substrate and nucleotide A,G,T or C); the y-axis shows the light intensity generated. The numbering of pyrograms corresponds to the haplotype numbers in Table 2. Note that all these samples were wild type for g.-168C > T.

The SSCP revealed that it was essential to get sequencing data for all samples for accurate genotyping. Thus, we used pyrosequencing, which is suitable for the amplification of this short region and provides exact sequence data for a large number of samples. Pyrosequencing was optimised and evaluated using genomic DNA prepared from blood from healthy adult volunteers. The initial assay was designed to use the forward strand but this approach was unsuccessful and the reading failed at the SNP g.-181A > C (Figure 1, Figure 3A and B left panel). Therefore, pyrosequencing was carried out on the reverse strand which generated clear pyrograms (Figure 3B right panel, Figure 4). Note that the sequence is given in reverse orientation.

### Polymorphism analysis of the EAAT2 promoter using pyrosequencing

Successful amplification was obtained in 209 samples (87.5% success rate). Failure of the remaining samples was likely due to low quality genomic DNA. Some of the samples were 22 years old and showed DNA degradation [29]. Overall in 89% of the samples the polymorphisms g.-200C > A;-181A > C were inherited together (Table 2). While the SSCP data indicates that the genotype distribution of these SNPs is in Hardy Weinberg Equilibrium (HW), the pyrosequencing results suggest the opposite (Table 3). Measures of LD (D’ and r^2^), the non-random association between alleles of different loci, are consistent with the SNPs being linked (Table 3). The analysis and interpretation of LD is difficult due to the lack of HW and the presence of only one mutation at the -168 loci. Haplotype predictions are also shown in Table 4. A 100% concordance was observed when compared with Sanger sequencing (n = 51 samples were sequenced with both methods).

**Table 2:** Distribution of genotypes in the sample cohort

**Genotype**	**-200C > A**	**-181A > C**	**-168C > T**	**Number & proportion**
**1**	C/C	A/A	C/C	42 (20%)
**2**	C/A	A/C	C/C	110 (53%)
**3**	A/A	C/C	C/C	34 (16%)
**4**	C/A	C/C	C/C	7 (3%)
**5**	A/A	A/A	C/C	4 (2%)
**6**	C/A	A/A	C/C	0
**7a**	C/C	A/C	C/C	9 (4.5%)
**7b**	C/C	A/C	C/T	1 (0.5%)
**8**	A/A	A/C	C/C	2 (1%)
**9**	C/C	C/C	C/C	0
n = 209				
**WT**	C = 0.54	A = 0.51	C = 0.997	**Allele frequency**
**MT**	A = 0.46	C = 0.49	T = 0.002	

Genotypes were identified by pyrosequencing (n = 209) and confirmed by Sanger sequencing (n = 51). WT – wild type; MT – mutant.

**Table 3:** **Hardy Weinberg equilibrium and LD variance for the three *EAAT2 *** **SNPs using pyrosequencing or SSCP**

	**Pyrosequencing**	**SSCP**
	**Hardy-Weinberg**	
**for -168**	p > 0.99	N/A
**for -181**	p = 0.0188	p = 0.4063
**for -200**	p = 0.0256	p = 0.3325
	**Lewontin’s D’**	
**-168 and -181**	0.49	N/A
**-168 and -200**	1	N/A
**-181 and -200**	0.94	1
	**r**^**2**^	
**-168 and -181**	0	N/A
**-168 and -200**	0	N/A
**-181 and -200**	0.79	0.99

Lewontin’s D’ and r^2^ both give ordinal measures of Linkage Disequilibrium (LD). Please note only one mutation was found at -168 making interpretation difficult for these associations.

**Table 4:** Predicted haplotype frequencies in the cohort using pyrosequencing or SSCP

**Pyrosequencing**										
**SNP**	**Haplotype**								**C allele**	**Total alleles**
-168 C > T	T	T	T	T	C	C	C	C	417	418
-181 A > C	A	A	C	C	A	A	C	C	204	418
-200 C > A	A	C	A	C	A	C	A	C	225	418
%	0.0	0.0	0.0	0.2	1.5	49.8	44.6	3.9		
					**SSCP**					
**SNP**	**Haplotype**				**C allele**	**Total alleles**				
-181 A > C	A	A	C	C	208	414				
-200 C > A	A	C	A	C	205	414				
%	0.2	49.5	50.2	0.0						

(Total number of ‘C’ alleles is indicated).

### Comparison of sample genotyping using pyrosequencing and SSCP

**Figure 5:**
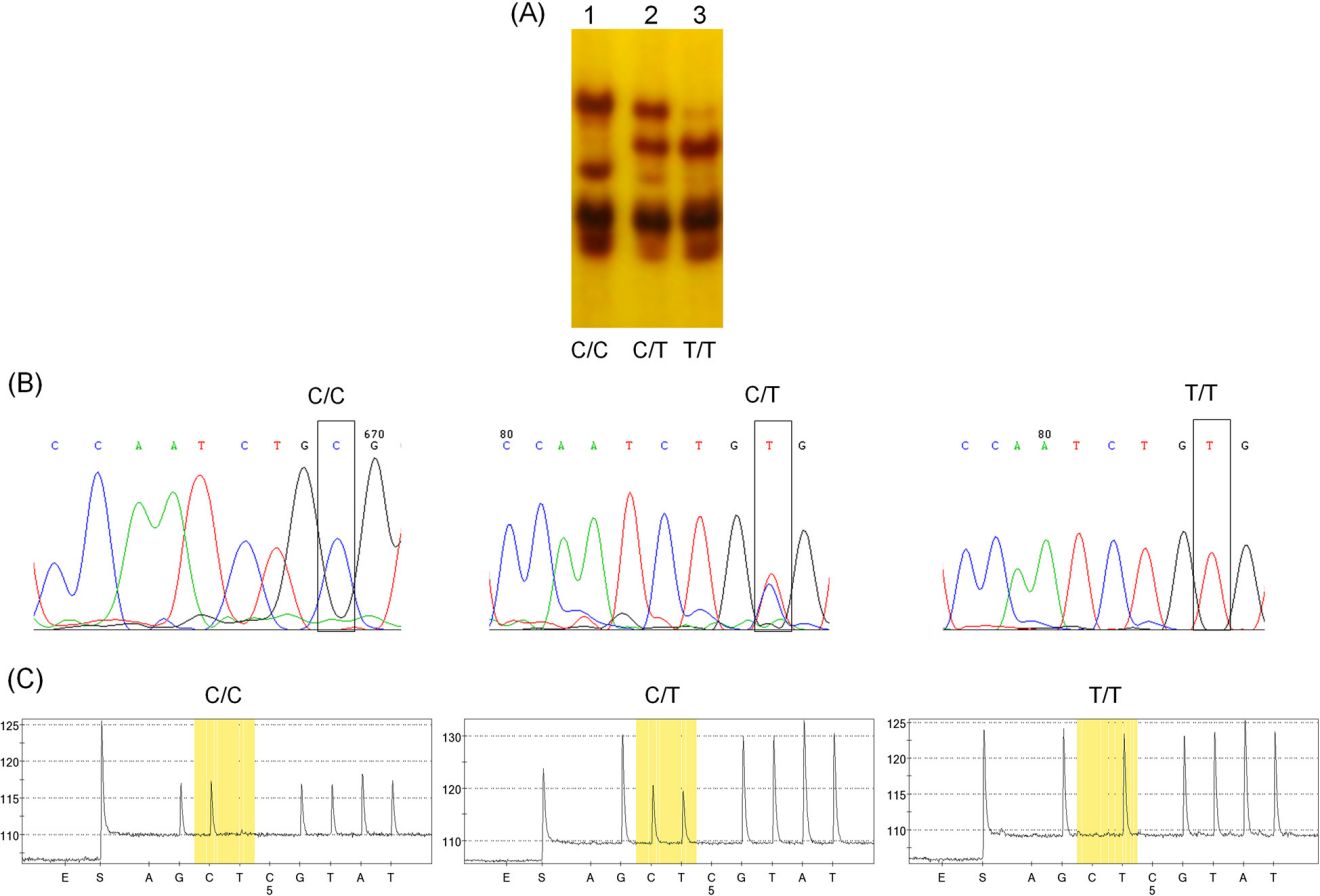
**Detection of an unrelated SNP, rs1835740, by SSCP, Sanger sequencing and pyrosequencing. (A)** Genotype of each sample determined by Sanger sequencing is shown at the bottom of each lane. **(B)** SNP is indicated in rectangles on the Sanger sequence. **(C)** The position of the SNP on the pyrogram is highlighted in yellow boxes.

All nine sequence combinations have been successfully amplified and pyrosequenced (Figure 4; Note that genotypes six and nine were only found in the adult control samples hence they are not presented in Tables 2, 5 and 6). 239 samples from newborn infants were initially used and 209 could be classified for SSCP and pyrosequencing. Because different samples failed to produce clear PCR products for SSCP and pyrosequencing, a total of 183 samples generated result with both genotyping methods. With SSCP a total of 29 samples (16%) were incorrectly genotyped (Table 5). While 51 samples were classified as homozygote wild type using SSCP, pyrosequencing revealed that 25% of these samples do not belong to this group (Table 5). There was surprisingly little error in the identification of the heterozygotes with SSCP and 96% of the samples were correctly genotyped. In contrast, 12 homozygote mutants (31%) were incorrectly identified (Table 5). We also genotyped a small number of samples (n = 15) that failed to produce a clear PCR product with the EAAT2F and EAAT2R primers (hence could not be used for SSCP, Figure 1) but resulted in clear pyrograms with the EAAT2PyroSeq1 primer. A second SSCP was carried out with EAAT2F and EAAT2PyroR primers (Table 6) and found that with these primers similar proportion (20% *versus* 16%) of samples were misclassified as with the EAAT2F and EAAT2R primers.To compare the concordance between SSCP and pyrosequencing for a single SNP, SNP rs1835740 was analysed in the same 239 samples. Three distinct SSCP patterns were observed for the different genotypes (Figure 5A) which were confirmed by a random Sanger sequencing (Figure 5B) and pyrosequencing (Figure 5C) of the whole cohort. The concordance rate between SSCP and pyrosequencing was 94% for this SNP.

**Table 5:** Comparison of genotypes identified by SSCP and pyrosequencing

**SSCP**	**Pyprosequencing**								
**Genotype**	**1**	**2**	**3**	**4**	**5**	**6**	**7**	**8**	**9**
**1. WT/WT** (n = 51)	**38 (74.5%)**	1 (1.9%)	0.0	0.0	4 (7.8%)	0.0	8 (15.6%)	0.0	0.0
**2. WT/MT** (n = 92)	0.0	**88 (95.6%)**	2 (2.2%)	0.0	0.0	0.0	0.0	2 (2.2%)	0.0
**3. MT/MT** (n = 39)	0.0	6 (15.3%)	**27 (69.2%)**	6 (15.4%)	0.0	0.0	0.0	0.0	0.0
**4.** (n = 1)	0.0	0.0	0.0	**1.0**	0.0	0.0	0.0	0.0	0.0
n = 183	n = 183								

For the SSCP EAAT2F and EAAT2R primers were used and pyrosequencing was done with EAAT2PyroSeq1 primer (Figure 1 and Table 1). The genotypes that were correctly identified by both methods are indicated in bold. The genotype numbers indicated under pyrosequencing corresponds to those used in Table 2.

**Table 6:** Comparison of genotypes identified by SSCP and pyrosequencing

**SSCP**	**PYROSEQUENCING**								
**Genotype**	**1**	**2**	**3**	**4**	**5**	**6**	**7**	**8**	**9**
**1. WT/WT** (n = 3)	**2 (66.7%)**	0.0	0.0	0.0	0.0	0.0	1 (33.3%)	0.0	0.0
**2. WT/MT** (n = 11)	0.0	**9 (81.8%)**	1 (9.1%)	0.0	0.0	0.0	1 (9.1%)	0.0	0.0
**3. MT/MT** (n = 1)	0.0	0.0	**1 (100%)**	0.0	0.0	0.0	0.0	0.0	0.0
n = 15	n = 15								

For SSCP EAAT2F and EAAT2Pyro primers were used and pyrosequencing was done with EAAT2PyroSeq1 primer. The genotypes that were correctly identified by both methods are indicated in bold.

**Figure 6:**
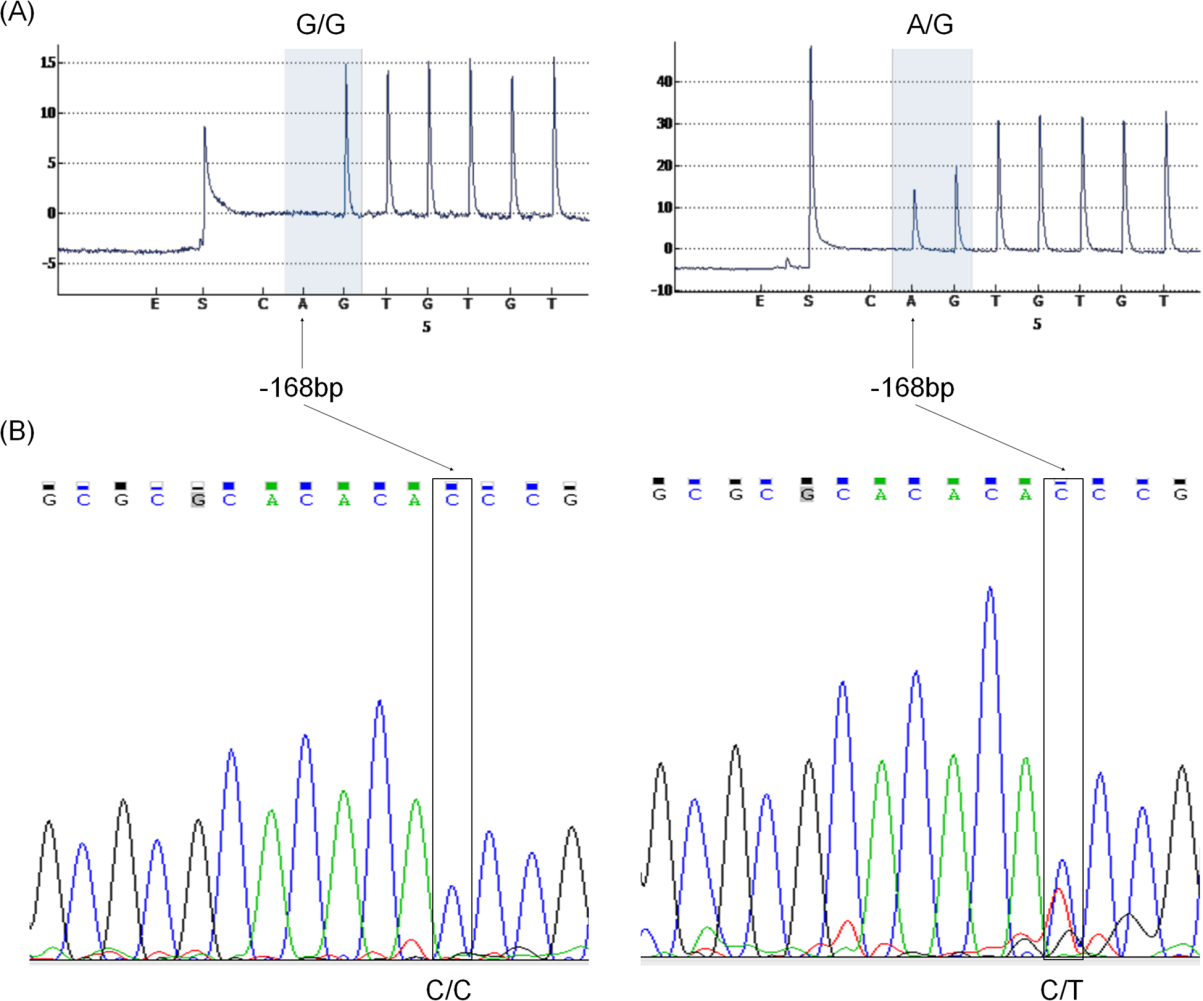
**Detection of the SNP in -168bp (g.-168C > T) in the *EAAT2 *promoter.** Pyrogram **(A)** and Sanger sequencing **(B)** of the homozygote WT and heterzygote samples.

While our investigation was underway, a SNP g.-168C > T was entered into the Database of Single Nucleotide Polymorphisms (dbSNP), through the 1000 Genomes Project [30] and was given a reference number of (rs116392274:C > T; Human Build 137). This nucleotide change is located in the EAAT2PyroSeq1 primer sequence (Figure 1) and thus it could not be observed in the pyrograms. However, using Sanger sequencing 51 samples were sequenced with EAAT2R (Figure 1) and in all of these samples only the C allele was observed at position -168bp. Furthermore, a pyrosequencing assay was developed to detect this g.-168C > T specifically (Figure 6). Of the analysed samples, 213 were wild type (C/C) and one sample was a heterozygote (C/T) for this SNP. The MAF was 0.0023 in our cohort.

## Discussion

### Identification of additional SNPs in the EAAT2 promoter

We identified a polymorphism at -200bp in the EEAT2 promoter, 19bp upstream of the previously reported and characterised polymorphism at -181bp (rs4354668:A > C or g.-181A > C) [11]. Our data indicates that these SNPs are in LD (Table 3). While our study was close to completion, the g.-200C > A was added to the NCBI SNP Database (1000 Genome Project, Human Build 137; rs111885243:C > A) confirming our sequencing and pyrosequencing data. The MAF in our predominantly Caucasian cohort for g.-200C > A and g.-181A > C is 0.46 and 0.49, respectively. The Global MAF available from the SNP Database are 0.39 and 0.41, respectively. More recently another SNP in the *EAAT2* promoter at position -168bp was added to the NCBI SNP Database (Human Build 137; rs116392274:C > T). In the 51 samples that we sequenced only the C allele was present. Furthermore, in the entire cohort (n = 214) only one T allele was found in a heterozygous form (Table 2). To date, these newly identified SNPs (g.-200C > A and g.-168C > T) have not been investigated in association studies or cited in the literature.

### SSCP is not sensitive enough to reliably distinguish between the various EAAT2 promoter genotypes

SSCP was used initially in this study because this method has previously been applied to genotype exactly the same region of the *EAAT2* promoter [11]. We used the same primers and PCR conditions as reported [11] but modified the SSCP running conditions that provided better separation of the DNA strands. Previously, approximately 2 h at a high voltage was used to resolve the amplicons. In contrast, in the current study the PCR products were resolved for 14 h at a relatively low voltage (150V) at a constant temperature (20°C). This allowed better separation and visualization of the ssDNA bands and lead to the identification of an additional genotype (Figure 2, Lane 4). Sequencing of several samples lead to the identification of g.-200C > A, which was not reported in a previous study of this region [11]. Our SSCP, pyrosequencing and Sanger sequencing highlighted that although four clear migration patterns can be seen (Figure 2, Lanes 1-4) several of the other variants (Figure 2, genotypes 5-9,) could not be identified by SSCP. Note that the reproducibility of SSCP was 100% for the samples that were used as controls (one sample from each of the three main genotypes were always run on each gel, in total n = 45 samples).

Studies using SSCP showed that the position of the substitution within a codon and the nucleotide itself can determine whether a SNP is detected [31]. A G to A or G to T nucleotide change at the second position of a codon caused a shift in ssDNA migration while failed to do so if it occurred in the first position [31]. In our case the SNP at -181bp is located on the second base, while the SNP at -200bp is on the first base of a codon. Furthermore, some nucleotide changes are detected at lower rates than others. For example, A to C transversions were detected at a higher rate (95%) compared to C to A transversions (82%) [31]. The SNP at -200bp is a C to A whilst the SNP at -181bp is an A to C transversion. It is also documented that some point mutations are not detected because of the nucleotide composition (e.g. A + T or G + C richness) of a DNA region being analysed [32]. Indeed, the *EAAT2* promoter is highly GC-rich (Figure 1). The amplicon used in both the previous study [11] and this study for SSCP analysis has a GC content of ~73%. Furthermore, some mutations may cause relatively small changes in electrophoretic mobility [33] and might remain undetected by SSCP [34-36]. These factors could explain that the SSCP patterns for the *EAAT2* promoter resemble that of a single SNP instead of multiple SNPs. However, the banding pattern does not fully correspond to the genotype of the SNP at -181bp. While genotypes 5 and 8 followed the -181bp SNP migration pattern, genotypes 6 and 7 resembled the migration of the -200bp SNP.

Based on the SSCP analysis, 25-31% of the WT/WT and MT/MT samples were mis-classified (Table 5 and 6). The previous study of the *EAAT2* promoter region [11] identified only three SSCP patterns in their cohort. However, considering the MAF of g.-200C > A and g.-181A > C in the population (0.46 and 0.49 in the current predominantly Caucasian cohort; 0.39 and 0.41 in the SNP Database), it is expected that some of the additional variants described here, should have been identified in the previous study (Table 4). Indeed, a similar allele frequency and LD levels are expected in Caucasian cohorts [37]. Furthermore, numerous subsequent studies [27,28,38,39] understandably continued with only investigating the association of this single SNP (rs4354668:A > C or g.-181A > C) with various diseases.

Many studies across different fields still use SSCP extensively as a genotyping method and about 1040 studies are listed on PubMed that used SSCP since 2010 to date. It is a simple, user-friendly, low cost method of SNP detection which does not require specialist equipment and can be adapted to a high-throughput format. It can work very effectively when a single SNP is investigated as we have demonstrated for an unrelated single SNP, rs1835740:C > T (Figure 5). However, our results also highlight that SSCP cannot always be used effectively when several SNPs are located in the target sequence. Although it is well recognised that a representative sample with distinct SSCP pattern needs to be sequenced to validate the method, it is also crucial that the entire sequence of the PCR product used for SSCP is scrutinised carefully. Generation of shorter PCR products for SSCP can sometimes help to uncover previously unnoticed variants [36,40]. If SSCP is used for genotyping (not for mutational screening) and all SNPs in the regions are known, covering some of them with primers may eliminate them from the SSCP pattern making the analysis of the remainders easier. The PCR products for the *EAAT2* promoter are already short, generating even shorter targets thus would not solve the problem seen with this particular target sequence but might offer solution for other troublesome targets.

### Pyrosequencing as an alternative to detect closely positioned SNPs

In our study g.-200C > A;-181A > C could simultaneously be analysed by pyrosequencing (Figures 3, 4 and Table 2). The detection limit of this method is dependent on how well the dispensation profile can be set up. This in turn depends on the nucleotide change within the SNP and the nucleotides adjacent to the SNP(s) [41]. Indeed, the latter caused problems in the genotyping of g.-181A > C using a primer in 5′-3′ orientation (Figure 3B, left panel). A four C mononucleotide repeat precedes this SNP and the non-linear light generation of the mononucleotide repeat made it impossible for the software to interpret the correct number of incorporated identical nucleotides [42,43] and as a consequence the assay failed at the g.-181A > C SNP (in 100% of the 96 samples analysed). This problem was overcome by re-designing the assay on the reverse strand and sequencing the nucleotide change prior to the C mononucleotide repeats (Figure 3B, right panel). Similarly, g.-168C > T was also sequenced on the reverse strand (Figure 1). Both pyrosequencing assays generated sequences immediately downstream of the primer (Figure 1, 3 and 6), which cannot be achieved with Sanger sequencing that lays a reading gap of 20-30 bp from the sequencing primer [44]. Pyrosequencing can only analyse a few positions simultaneously [41], which was the main reason for developing two separate assays to detect g.-200C > A;-181A > C and g.-168C > T (Figure 1). This approach resulted in clear and distinguishable pyrograms for each genotype for each assay. g.-168C > T was found in a heterozygote form in one infant with no clinical evidence of white matter injury (Rajatileka et al. unpublished observation). The MAF of the g.-168C > T (0.0023 in our study and 0.017 on the SNP Database) is very low in the general population which makes it challenging to assess in association studies. SNP-SNP interactions have been suggested to have a great impact on unveiling the underlying mechanism of complex diseases [45]. Thus, future clinical investigations of the impact of g.-200C > A;-181A > C on the promoter function of *EAAT2* and their association with various diseases will need to be assessed simultaneously.

Currently, the detection of g.-200C > A;-181A > C cost £1.79 and £1.43 by pyrosequencing and SSCP, respectively. For pyrosequencing the cost includes all reagents and a charge for the use of the pyrosequencer. For SSCP the cost was calculated from the reagents and Sanger sequencing of 10% of the samples. Following PCR amplification, the pyrosequencing required 1 h preparation time and 21 min run time for the two SNPs for 96 samples. For a single SNP (such as rs1835740:C > T) the run time is usually ~10 min for 96 samples. In contrast, SSCP analysis of 100 samples requires 2-3 h post-PCR preparation time, 12-16 h gel electrophoresis and 0.45-1.5 h silver staining. In addition, at least 10% of the samples need to be prepared for Sanger sequencing. Whilst pyrosequencing provides a less labour intensive, low cost and high throughput platform to genotype samples, in laboratories with no access to this facility SSCP may be used reliably for genotyping if *(i)* all mutations in the region are known, *(ii)* the SSCP genotype readout is validated by another method, and *(iii)* in case of two SNPs in the region the indicative bands for both mutations are clearly and easily distinguishable.

## Conclusion

Our data suggest that SSCP cannot always detect reliably several closely located SNPs. Furthermore, caution is needed in the interpretation of the association studies linking only one of the co-inherited SNPs in the EAAT2 promoter to human diseases.

## Competing interest

The authors declare that they have no competing interests.

## Authors’ contributions

SR designed and carried out all experimental work and data analysis. DO carried out the statistical analysis. KL and DH arranged access to the adult and newborn clinical samples, and contributed to clinical study design. KL obtained research ethics, NHS R&D permissions, University of Bristol research sponsorship for use of human tissue and consenting processes. MW assisted with the pyrosequencing analysis. EM and AV advised on experimental design. SR and AV wrote the manuscript and all authors reviewed the manuscript prior to submission. All authors read and approved the final manuscript.
